# Reduction in energy for electrochemical disinfection of *E. coli* in urine simulant

**DOI:** 10.1007/s10800-019-01292-4

**Published:** 2019-03-06

**Authors:** Akshay S. Raut, Charles B. Parker, Ethan J. D. Klem, Brian R. Stoner, Marc A. Deshusses, Jeffrey T. Glass

**Affiliations:** 1 Department of Electrical and Computer Engineering, Duke University, Center for WaSH-AID, Durham, NC 27708 USA; 2 RTI International, Discovery-Science-Technology Division, Research Triangle Park, NC 27709 USA; 3 Department of Civil and Environmental Engineering, Duke University, Durham, NC 27708 USA

**Keywords:** Liquid waste remediation, Boron-doped diamond, Electrochemical disinfection, Urine treatment

## Abstract

**Abstract:**

We report the development of novel modes of operation for electrochemical disinfection of *E. coli* in human urine simulant with an aim to minimize the energy required for disinfection. The system employs boron-doped diamond electrodes and will be part of an energy neutral, water and additive free outdoor toilet being developed for use in developing countries. Disinfection had been previously demonstrated with voltage being continuously applied to the electrode until disinfection was achieved. In the present study, a new pulsed mode of operation is investigated. This includes a continuous on mode, where oxidants are generated until disinfection is achieved, a single cycle mode, where oxidants are generated for a fixed time and the water is circulated so allow already generated oxidants to disinfect, and a pulsed mode with different duty cycles, which is like the single cycle mode but with multiple cycles. Disinfection was achieved with pulsed mode operation with a 68% energy reduction compared to the continuous on mode. Energy saving was most likely achieved by lengthening the contact time of the disinfectant with the bacteria and increased generation of non-chlorine disinfecting oxidants.

**Graphical abstract:**

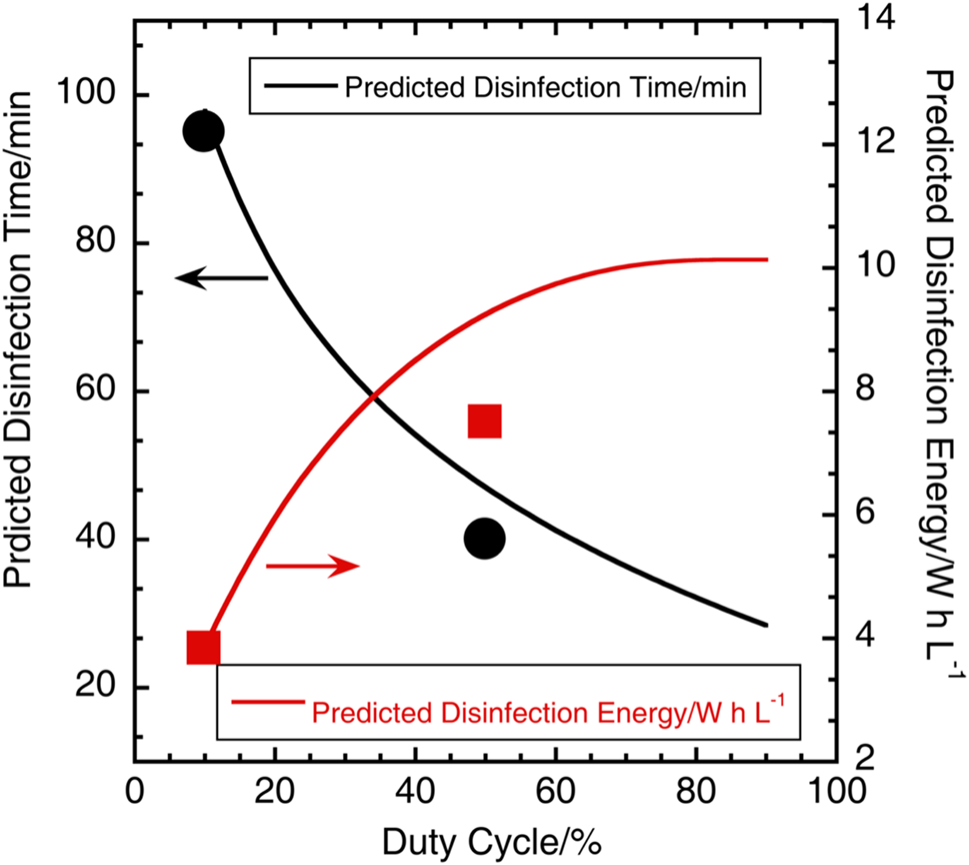

## Introduction

According to a World Health Organization (WHO) report [1], 2.6 billion people on the planet do not have access to adequate sanitation. In the less developed world where the problem is most acute, 760,000 children under the age of five die every year due to diarrhea resulting from poor sanitation [2]. Globally, there are 1.7 billion cases of diarrheal disease every year [2]. Improved sanitation is expected to drastically improve the health and quality of life of people in these regions [3]. Addressing the critical sanitation needs of communities in these regions requires wastewater and sewage treatment techniques that can function effectively despite significant infrastructure constraints, such as no or intermittent electrical power, limited supply of fresh water, and low availability of chemical reagents.

Separation of human waste from sources of water used for drinking and cleaning is necessary to reduce disease caused by poor sanitation. This requires changing our approach to toilet design or, as coined by the Bill & Melinda Gates Foundation, “reinventing the toilet” [4]. The research reported here is part of a project to develop an energy-neutral, additive-free, and water-free toilet with integrated treatment for point of use sanitation, aimed for the developing world, and costing less than $0.05 per user per day.

Disinfection of liquid waste can be achieved by use of various techniques such as chlorination, UV disinfection, filtration/membrane processes, thermal treatment, and electrochemical treatment [5]. Electrochemical disinfection of liquid waste refers to the elimination of microorganisms present in the liquid by use of suitable electrodes to initiate pathogen-killing electrochemical processes in the waste stream undergoing treatment. The electrochemical disinfection method reported here produces reactive oxygen species (ROS) such as ozone, hydrogen peroxide and hydroxyl radicals as well as hypochlorous acid/hypochlorite ions as parallel reactions to oxygen evolution at the anode [6–8]. The chlorine-containing species (CCS) are derived from chloride ions present in urine. These ROS and CCS are responsible for inactivation of pathogens, thereby reducing the spread of disease causing agents. Although electrochemical techniques have been investigated for inactivation of pathogens [9–18] and have been shown to be more effective at bacterial inactivation than direct chemical chlorination or ozonation [19], they were not specifically developed for disinfection of undiluted human waste. Moreover, energy efficiency was not a key focus in these previous studies.

Electrochemical disinfection systems can be made more energy efficient using electrodes with a higher oxygen evolution overpotential, so that most of the applied current is utilized for the generation of disinfecting oxidants rather than oxygen generation. Thus, a high overpotential can improve energy efficiency if the electrode can produce ROS and CCS species without simultaneously producing unwanted side reactions. In addition, the electrode must be chemically stable in the electrolyte of interest. Boron-doped diamond (BDD), which has been used in this study, possesses both of these attributes in aqueous electrolytes and thus is a promising electrode for wastewater disinfection [20, 21]. For BDD electrodes, hydrogen evolution occurs at − 1.25 V versus a standard hydrogen electrode (SHE) and oxygen evolution occurs at + 2.3 V versus SHE [22]. It has been shown that various disinfecting oxidants such as chlorine, ozone, and hydroxyl radicals are generated at anodic potentials below 3 V at BDD electrodes in NaCl solution [23].

In a previous publication [24], we reported that the rate of generation of CCS and the rate of inactivation of *E. coli* in human urine simulant at the BDD electrode was a function of the applied electrode voltage. The energy required to meet WHO disinfection requirements was calculated from the applied voltage, measured current and the volume of liquid being processed [25, 26]. We observed that there was a trade-off between the energy per unit volume of liquid required for disinfection and the time required for disinfection per unit volume of liquid [24]. For field implementation, the electrode voltage must be chosen such that both the time required for disinfection and the energy consumed meet the constraints for the application of interest. Our toilet design requires the liquid waste subsystem to process approximately 8 L of urine per day (corresponding to approximately five users per day) [27] using energy provided from low cost solar panels or recovered from the combustion of the solid waste. With the goal of minimizing energy utilization, novel operating modes are required. In this paper, we describe an operation scheme that minimizes energy consumption by varying the applied electrode voltage to increase germicidal efficiency while keeping the disinfection time and efficacy within design constraints.

## Experimental setup and procedure

### Experimental setup

**Fig. 1:**
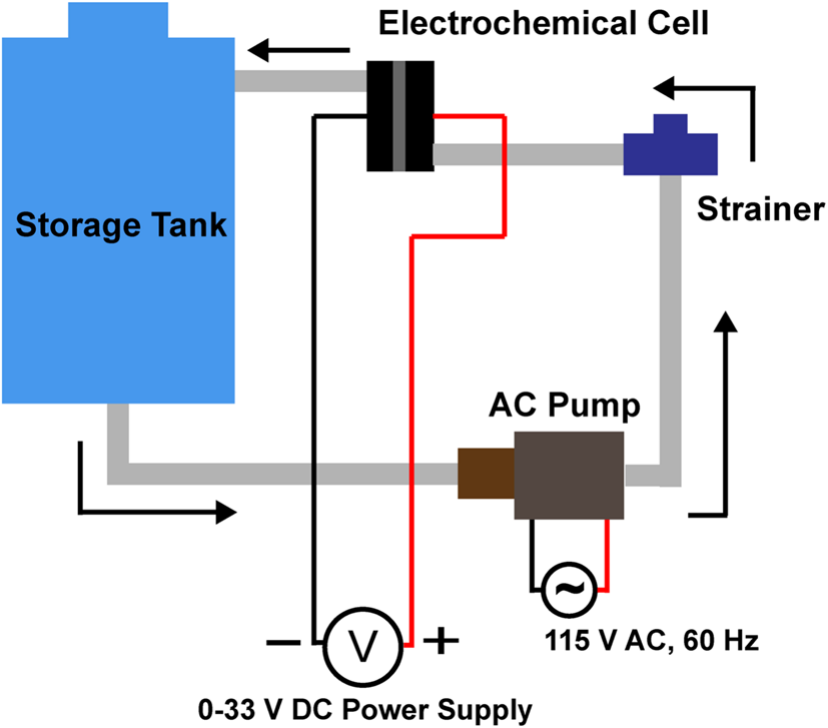
Schematic of disinfection system setup. The arrows indicate the direction of liquid flow. The opening on top of the storage tank is used to introduce the liquid into the system and also to draw samples

An electrochemical cell (Advanced Diamond Technology’s Diamonox 40 system) was integrated with an 8 L storage tank, drain valve, strainer, and AC pump. A schematic of this assembly is shown in Fig. 1. Details about the electrodes have been reported in previous publications [24, 28]. Briefly, the anode was a 2 µm boron-doped nanocrystalline diamond film on a niobium substrate and the cathode was a tungsten plate. The area of each electrode was 42 cm^2^ and the spacing between the electrodes was 2 mm. The volume of liquid being treated was initially held in the storage tank. The liquid was returned to the tank after every pass through the electrochemical cell. During disinfection experiments, the liquid was pumped continuously at a rate of 2 L min^−1^, the rate recommended by the manufacturer, from the storage tank, through the electrochemical cell, and returned to the storage tank, thus forming a closed loop. A Sorensen XHR 33–33 (0–33 V, 0–33 A) power supply, which measures the current for a nominal voltage, was used to power the electrodes. As noted below, total chlorine was measured as an indicator of the quantity of oxidants in the system, however, the specific oxidants present were not measured, as synthetic urine is a highly complex electrochemical system. Further, we did not measure chemical oxygen demand (COD), as other work by the authors determined that it is necessary to use much more energy to reduce COD measurably than the energy required to achieve electrochemical disinfection [29, 30].

### Synthetic excreta

To study inactivation of bacteria in human waste, synthetic urine was used as the simulant. The recipe (Table 1) for synthetic urine was adopted from those suggested in prior literature [31, 32]. Synthetic urine prepared this way was sterilized in an autoclave at 121 °C and 2 atm for 20 min. After cooling to room temperature, a suspension of *E. coli* was mixed into the sterile synthetic urine, referred to as spiking the synthetic urine, as discussed below.

**Table 1:** Composition of human urine simulant

Chemical	Quantity (per liter of deionized water)
Na_2_SO_4_	2.34 g
KCl	3.88 g
NH_4_OH (28–30%)	1.33 mL
MgSO_4_·7H_2_O	0.49 g
Na_2_HPO_4_	1.85 g
H_3_PO_4_ (85% aqueous solution)	0.25 mL
NaOH	0.14 g
NaCl	9.93 g
Trisodium citrate 2H_2_O	0.47 g
Urea	30.3 g
Creatinine	1.47 g
Hippuric acid	0.54 g
Uric acid	0.50 g

### Indicator organism

*Escherichia coli* (*E. coli*) is commonly used as an indicator organism in disinfection studies [11, 12, 15, 19, 33–35]. The indicator organism used in this study was *E. coli* K12 (ATCC #10798). It was grown in a broth containing 10 g L^−1^ of tryptone, 5 g L^−1^ of yeast extract, and 10 g L^−1^ of NaCl in deionized water. The broth was sterilized as above. After cooling to room temperature, the *E. coli* broth was inoculated from a stock culture using standard aseptic techniques to avoid cross-contamination [36]. The culture was incubated at 37 °C for 24 h. After incubation, 200 mL of culture was mixed with 1.8 L of sterile synthetic urine, yielding 2 L of spiked synthetic urine. The initial cell concentration was 10^10^ colony-forming units per 100 mL (CFU 100 mL^−1^).

### Sample plating

The samples drawn during the experiment were diluted serially in sterile 8 g L^−1^ NaCl solution to obtain viable bacteria counts according to standard protocols [37]. For each dilution, 100 µL was plated onto 100 mm petri dishes prepared with RAPID’E. coli 2 agar (Bio-Rad Inc.), a chromogenic medium for enumeration and differentiation of generic *E. coli*. The serial dilution and plating of three samples from each experiment were duplicated to confirm the cell counts. The plates were incubated at 37 °C for 24 h after which the CFUs on each plate were counted and reported as CFU per 100 mL.

### Total chlorine measurement

Total chlorine was measured with a DR890 colorimeter (Hach) and the *N,N*-diethyl-*p*-phenylenediamine (DPD) method (Hach test #8167). The volume of each sample was 10 mL. The test is approved by the United States Environmental Protection Agency and detects the presence of free chlorine (hypochlorous acid/hypochlorite ion) and combined chlorine (monochloramine and dichloramine) [38].

### Testing procedure

**Fig. 2:**
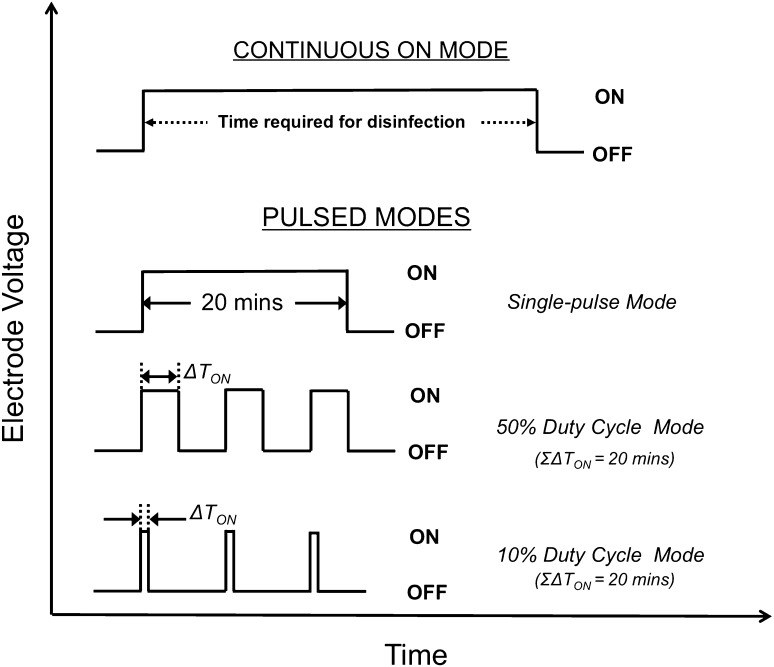
Different modes of applying electrode voltage with the goal of minimizing energy consumption during electrochemical disinfection of synthetic urine spiked with *E. coli*

**Fig. 3:**
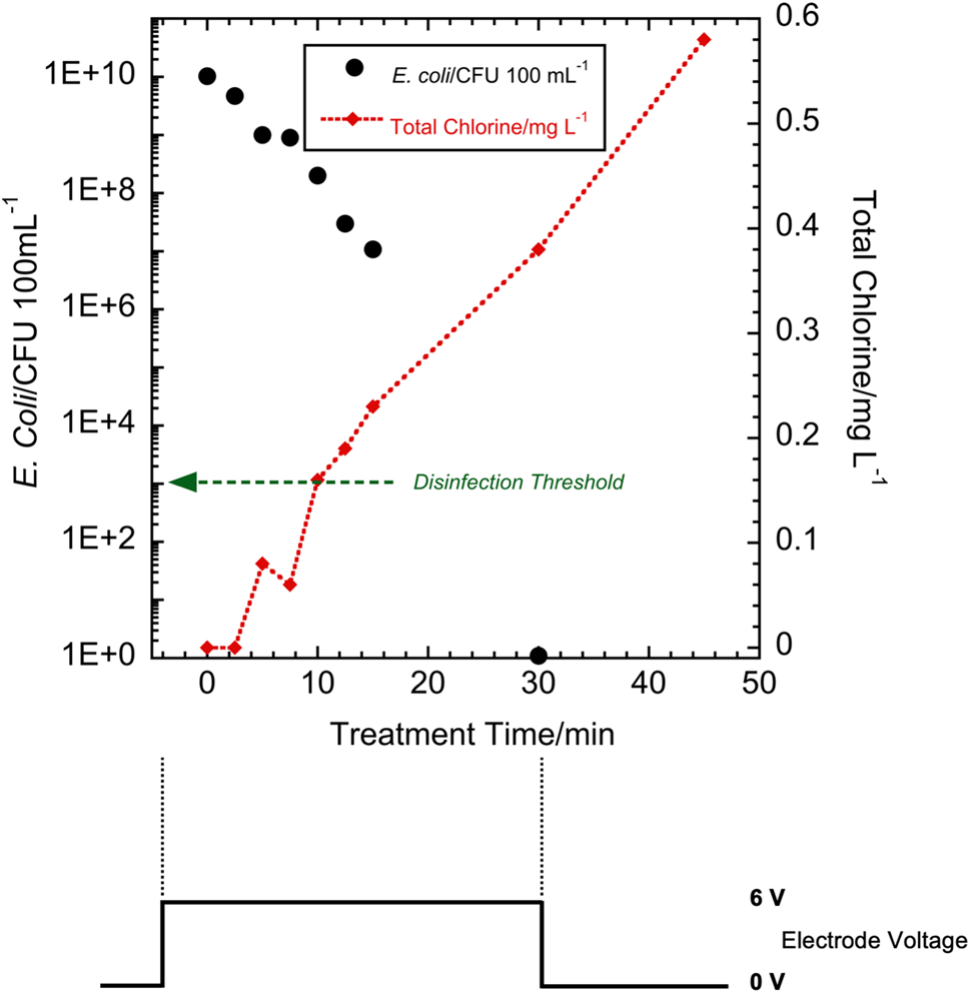
(Upper) *E. coli* CFU count and total chlorine concentration versus treatment time for continuous ON mode operation at 6 V. (Lower) nominal applied electrode voltage versus time

At the initiation of the experiment, the *E. coli-*spiked synthetic urine was introduced into the storage tank. Thereafter, the liquid recycle pump was turned on and the spiked synthetic urine flowed through the electrochemical cell loop. This also achieved effective mixing of the storage tank (mixing time was about 90 s, results not shown). The first sample was drawn from the storage tank with a sterile pipette prior to application of a voltage to the electrodes. The voltage was then applied to the electrodes and samples were drawn in a similar manner with a new sterile pipette at each specified time during the experiment. The electrode voltage and current were noted from the power supply display each time a sample was drawn. In the present study, three pulsed modes of operation were investigated to determine if energy consumption could be minimized while achieving disinfection. First was the single cycle mode, where the electrode voltage was turned off after 20 min, but the pump was kept on for continued mixing. In the second mode, the electrode voltage was applied intermittently as a square wave with a 50% duty cycle. The third mode was a square wave with a 10% duty cycle. The amplitude of the voltage was 6 V for all modes. After a startup period of less than 15 s, the current remained stable at 3.7 A. These modes of operation are illustrated in Fig. 2. For reference, in our previous publication [24], energy consumption to achieve disinfection was determined for a constant and continuous electrode voltage; the electrode voltage was kept on until the WHO disinfection threshold was achieved (defined as *E. coli* concentration < 10^3^ CFU per 100 mL). We refer to that mode of operation as the continuous ON mode and a typical disinfection run is shown in Fig 3.

In the single-pulse mode at 6 V, the electrode voltage was turned OFF after 20 min and corresponding bacteria count and total chlorine levels were measured for a total of 100 min at 2 min intervals for the first 20 min, then at 10 min intervals thereafter. Although the electrode voltage was turned OFF after 20 min, the pump was kept on for 100 min to allow for mixing and reaction of oxidants, and ultimately stabilization of the CFU count.

In the 50% duty cycle pulsed mode operation, electrode voltage was applied in the form of a pulse with 4 min ON time and 4 min OFF time. A total of five pulses was applied for a total ON time of 20 min, which is the same as in the case of the single-pulse experiments. The pump was kept on until the time a spike in total chlorine concentration was detected, 105 min, to allow for mixing and reaction of oxidants.

In the 10% duty cycle pulsed mode operation, electrode voltage was applied in the form of a pulse of 48 s ON time and 432 s OFF time, i.e., a period of 8 min. A total of 25 pulses were applied, for a total ON time of 20 min, which is the same as in the case of the single-pulse mode and 50% duty cycle pulsed mode. The pump was kept on for 185 min to allow for mixing and reaction of oxidants.

The specific energy required for disinfection is defined per the equation in Ref. [26]:

1 $$E=\frac{{V \times I \times t}}{L}$$

where *E* is the energy required for disinfection (Wh L^−1^); *V* is the electrode voltage (V); *I* is the current (A); *t* is the time required for disinfection (h); *L* is the treated volume (L).

The energy consumed by the pump is not included in the equation above.

## Results

### Single-cycle mode

**Fig. 4:**
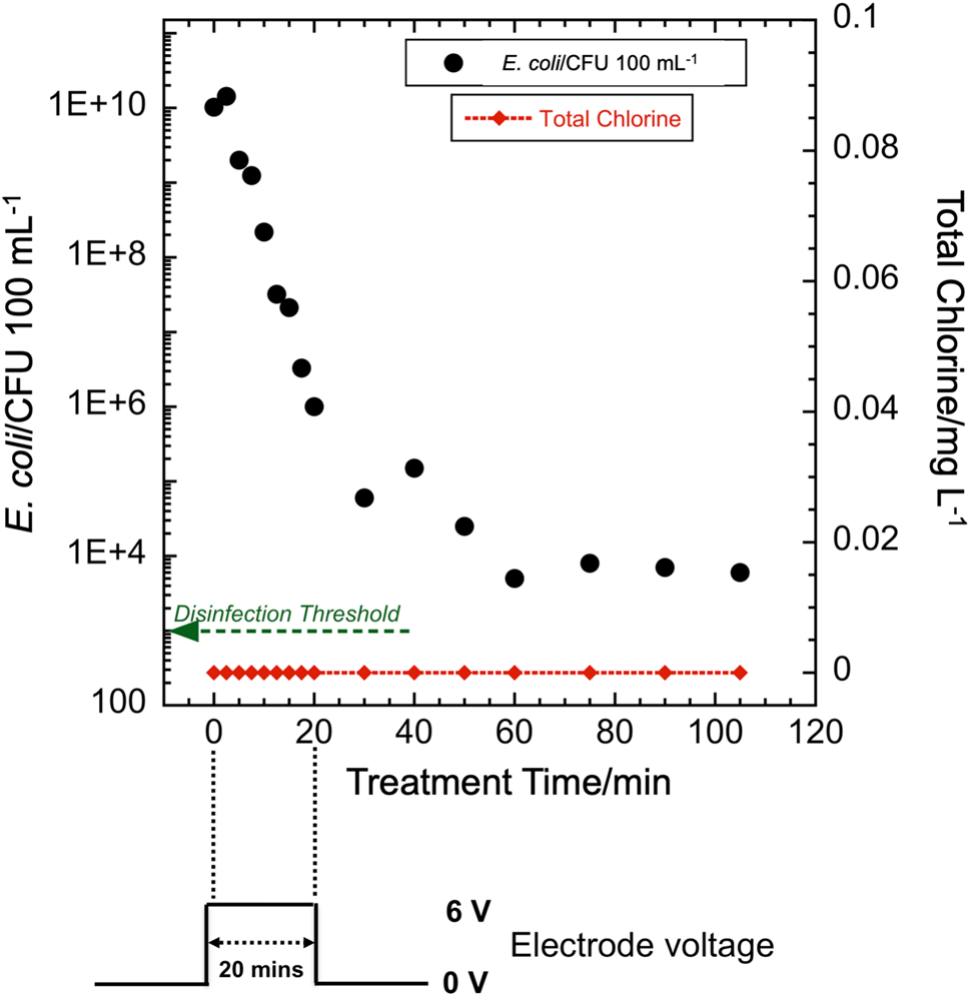
(Upper) *E. coli* CFU count and total chlorine concentration versus treatment time for single-cycle mode operation at 6 V. (Lower) nominal applied electrode voltage versus time

Figure 4 shows that *E. coli* count (CFU per 100 mL) decreased by approximately six orders of magnitude in 100 min, but it did not drop below the WHO [3] recommended level of 10^3^ CFU per 100 mL. The total chlorine concentration never rose above the detection limit (0.01 mg L^−1^) during these experiments. Total chlorine (sometimes referred to as “residual chlorine”) in the form of free and combined chlorine generally appears after all the demand for chlorine from the organics and nitrogen compounds has been met [38]. Incomplete disinfection in the case of the single-pulse test indicates that there is still demand for the chlorine and explains the absence of detectable total chlorine.

### 50% Duty cycle pulsed mode

**Fig. 5:**
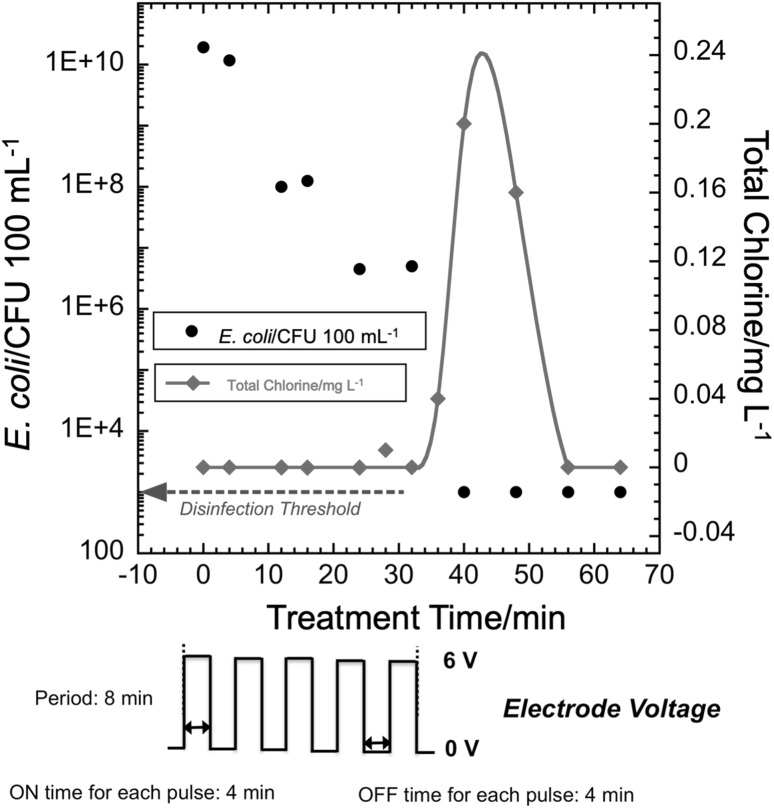
(Upper) *E. coli* CFU count and total chlorine concentration versus treatment time for 50% duty cycle pulsed mode operation at 6 V for disinfection of human urine simulant. (Lower) nominal applied electrode voltage versus time (Red line is a guide to the eye). (Color figure online)

In contrast to the single-pulse tests, the *E. coli* count fell below the WHO disinfection threshold for the 50% duty cycle tests (Fig. 5). This occurred in approximately 35 min of total time, including approximately 17.5 min of total ON time applied in 4 min pulses. An increase, or spike, in the total chlorine concentration was observed at approximately the same time that the CFU count fell below the disinfection threshold. This is consistent with the explanation that total chlorine rises when demand ceases.

### 10% Duty cycle pulsed mode

**Fig. 6:**
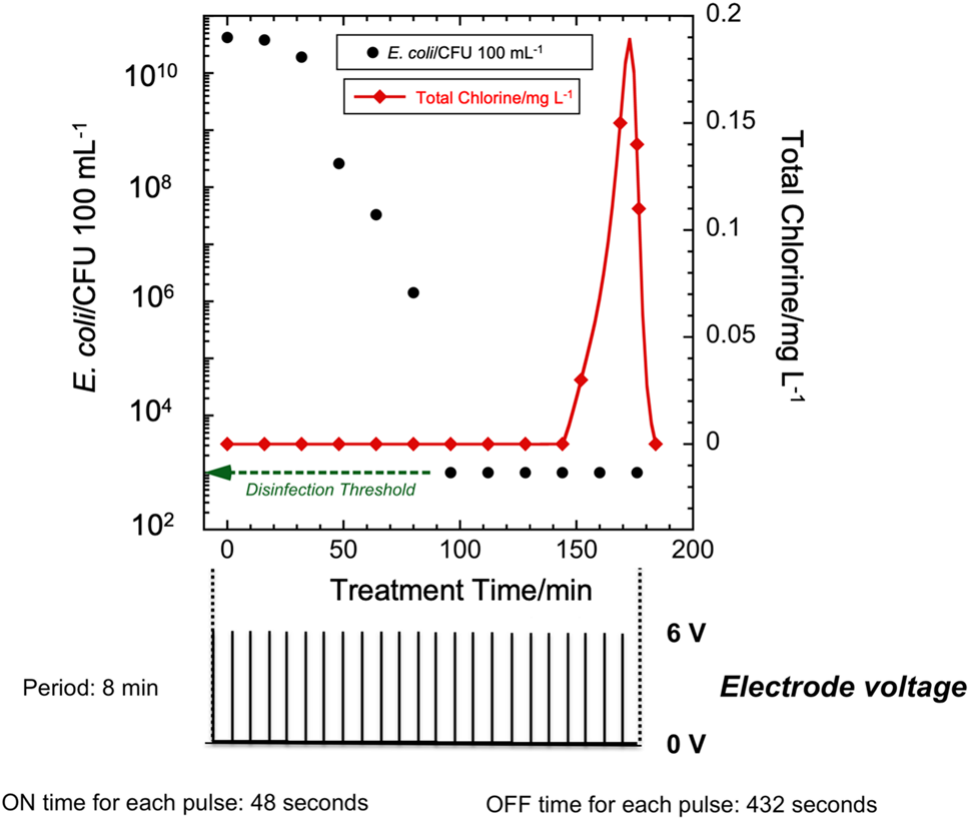
(Upper) *E. coli* CFU count and total chlorine concentration versus treatment time for 10% duty cycle pulsed mode operation at 6 V for disinfection of human urine simulant. (Lower) nominal applied electrode voltage versus time (Red line is a guide to the eye). (Color figure online)

As seen in Fig. 6, during the 10% duty cycle experiments, the *E. coli* count fell below the WHO disinfection threshold in 90 min of total time, which included approximately 9 min of total ON time delivered in 48 s pulses every 8 min. Just as in the case of the 50% duty cycle pulsed mode, a spike in total chlorine concentration was observed. However, the spike appears approximately 75 min after the *E. coli* count drops below the disinfection threshold.

## Discussion

### Disinfection and chlorine evolution in various modes of operation

Chlorine demand arises from the bacteria and components of the urine. It consumes chlorine and other reactive species generated during the electrolysis period. In the continuous ON mode, chlorine was simultaneously generated and consumed. This simultaneous production and consumption of chlorine is analogous to periodic dosing with chlorine [39, 40]. The electrochemical cell is expending energy during the entire disinfection process. In contrast, during the pulsed operating modes, the oxidants produced continued disinfection during the OFF time when no energy was expended and no new chlorine was generated. This OFF time disinfection is aided by continued mixing of the liquid undergoing treatment, enabling the chlorine and reactive species to interact with more of the *E. coli*. Disinfection requires a finite contact time between the chlorine and the bacteria. The pulsed modes allow additional contact time with no additional energy input. Thus, energy efficiency is increased by turning the voltage off and allowing previously generated reactive species to react with *E. coli*. The contact time required for disinfection depends upon both the *E. coli* bacteria and the oxidant concentrations [41, 42].

In previously reported experiments, the WHO disinfection threshold was achieved in 30 min for 2 L of urine for the continuous ON mode [24]. In the present work, for the 20 min single-pulse mode (Fig. 4), *E. coli* CFU counts did not decrease below the WHO disinfection threshold of 10^3^ CFU per 100 mL and total chlorine remained below the detection limit. The decrease in bacteria concentration suggests that chlorine was generated, but it did not reach a detectable residual value, as it was rapidly consumed. Although the WHO disinfection threshold was not achieved with 20 min of treatment at 6 V in this mode, there was a 6-log reduction in *E. coli* count. This indicates that the chlorine is consumed by the disinfection process and no detectable chlorine species remain. It also may indicate that other oxidants, such as hydroxyl radicals and ozone, have an impact on disinfection [7]. Subsequent experiments determined whether multiple pulses with the same ON time as a single-pulse mode would yield the required level of *E. coli* inactivation while reducing the energy required for disinfection.

For the 10% duty cycle pulsed mode at 6 V, the WHO disinfection threshold was achieved in approximately 100 min and a spike in total chlorine concentration was observed at 175 min. In contrast, for the 50% duty cycle mode at 6 V, disinfection below the WHO threshold was achieved in 40 min and a spike in total chlorine concentration was observed at approximately 40 min, i.e., approximately the same time disinfection was achieved. The total ON time in the 10% duty cycle mode (10 min) to achieve disinfection was much less than that in the case of 50% duty cycle mode (20 min). This indicates that along with the effect of chlorine, longer contact time with the *E. coli* and the effect of other disinfecting oxidants could be playing a role in making the 10% duty cycle mode a more energy efficient mode of operation.

### Effect of combined chlorine and other oxidants

In this study, there was demand on the free chlorine from the free ammonia and ammonium in the urine as well as the *E. coli*. Free ammonia is produced from the oxidation of urea [43] in urine, which is a side reaction to the generation of free chlorine. Hence the lifetime of free chlorine in this system is small until the chlorine demand is met.

In previous experiments (not shown), it was observed that the chlorine existed primarily as combined chlorine (monochloramine and dichloramine) during disinfection of the urine simulant. In the pH range of the urine simulant (approximately pH 8), the combined chlorine is mostly in the form of monochloramine, which is typically a weaker disinfectant [44, 45] than free chlorine. This will impact the disinfection rate and the expected CT factor for disinfection, as discussed below.

Germicidal efficiency of disinfectants depends upon the CT factor, which is defined as the product of the concentration (in mg L^−1^) of disinfectant and the contact time (in minutes) with the pathogen of interest [42, 46]. CT values for common disinfectants for various pathogens are generally provided for demand-free systems. In a demand-free system, the CT value of monochloramine for inactivation of *E. coli* has been shown to be 64 mg min L^−1^, whereas that of free chlorine is 0.04–0.92 mg min L^−1^ [46]. In the present work, there was no indication of the chlorine demand being met since the residual chlorine concentration detected after disinfection decayed over time. Nevertheless, CT values were calculated to allow comparison with values reported in the literature.

CT values were calculated by means of a formula for discrete time sampling [39] for both continuous ON mode and pulsed modes for the values of total chlorine detected in the continuous ON and pulsed mode tests. For pulsed modes, the CT values were calculated from the time the total chlorine spike was measured. For continuous ON mode, the CT value was 5.07 mg min L^−1^. For the 50% duty cycle pulsed mode, the CT value was 1.1 mg min L^−1^. Finally, for the 10% duty cycle, the CT value was 0.1 mg min L^−1^. These values are much lower than the value required for disinfection by monochloramine alone [46]. This suggests that disinfection was due to a combined action of monochloramine and other oxidants, such as hydroxyl radicals and ozone, with different germicidal potency. While chlorine was not measured, the presence of monochloramine means that it was generated and reacted immediately. The overall inactivation of *E. coli* by other disinfecting oxidants, such as hydroxyl radicals, ozone, and peroxide, may be impacted because of both different responses to pulsing in their generation rate and contact time needed for disinfection. Their relative contributions towards inactivation of *E. coli* are the subject of future work.

### Effect of contact time

**Fig. 7:**
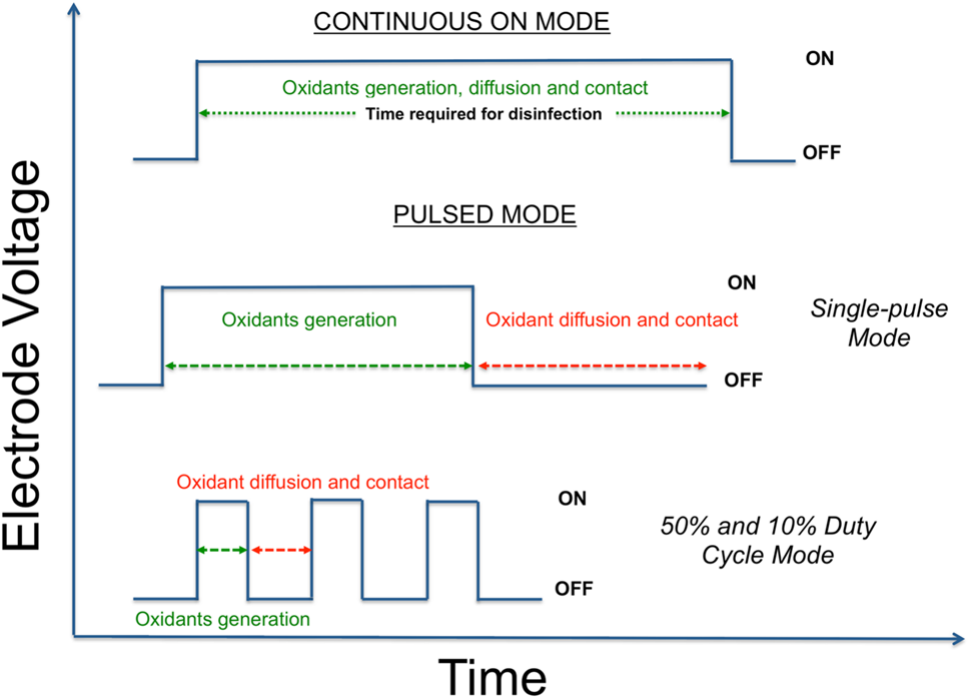
Description of disinfection process for the different modes of operation. The OFF time during the different pulsed modes provided greater contact time for the disinfectants with the bacteria

While a shorter ON time may generate a lower concentration of oxidants, experiments such as those reported in Figs. 5 and 6 demonstrate that OFF times allow for mixing and reaction. For a given CT value, higher T and lower C are more effective than the opposite [38]. Thus, as illustrated in Fig. 7, varying duty cycle in the pulsed mode offers a trade-off between oxidant concentration and contact time. This likely played a role in making the smaller duty cycle mode more energy efficient.

### Possible effect of electric field

It is possible that there is also direct disinfection from the electric fields in the electrochemical cell. Li et al. [40] observed that disinfection rate in an electrochlorination system was much higher than chemical chlorination for similar CT values. They concluded that disinfection in electrochemical systems could involve not only electrochemically generated chlorine species, but other mechanisms such as electroporation as well [40]. In the past, high voltage (12–30 kV cm^−1^), short duration (0.7–300 µs) electric field pulses have been shown to inactivate *E. coli* in food through direct mechanisms such as electroporation [47, 48]. In the present study, at 6 V electrode voltage and a spacing of 2 mm, the electric field was 30 V cm^−1^ with the shortest pulse duration of 48 s. Although the electric field strength is much lower and the pulse duration much longer in the present study as compared to the literature, pulsing of the electric field may favorably affect the disinfection rate through the electroporation mechanism. This is an area for future investigation.

### Disinfection energy

The energy consumed during the electrochemical disinfection process was calculated from Eq. 1. In the calculation, the energy consumed by the pump was not included and time *t* was the time taken as the time for the *E. coli* count to drop below the WHO disinfection threshold. For the pulsed modes, the electrode voltage was not on for the entire duration, *t*, but only during the ON time of the pulse. *t* is thus the product of ON time per pulse and number of pulses required to reduce *E. coli* count to the disinfection threshold. Table 2 shows the total cell voltage ON time, disinfection time and energy for each of the operating modes. The energy consumed during the 10% duty cycle pulsed mode operation is 68% less than that in continuous ON mode. On the other hand, as expected, the total time required for disinfection is higher in the pulsed mode as compared to the continuous mode.

**Table 2:** Total electrode voltage ON time, disinfection energy and time, CT value for different operating modes

Mode	Total cell voltage ON time for disinfection (min)	Energy for disinfection (Wh L^−1^)	Time for disinfection (min)	CT value (mg min L^−1^)
Continuous ON	30	11.18	30	5.0
Pulsed (50% duty cycle)	17.5	6.64	35	1.1
Pulsed (10% duty cycle)	9	3.58	90	0.1

### Effect of duty cycle on chlorine generation rate

Li et al. [40] reported a formula derived from Sawyer et al. [8] for obtaining the concentration of chlorine from applied current and hydraulic retention time inside an electrochemical cell. It is as follows:

2 $${C_{{\text{Cl}}}}=\frac{{35500 \times I}}{{F \times V}}$$

where *C*_Cl_ is the concentration of chlorine (mg L^−1^); *I* is the cell current (A), *F* is the Faraday’s constant; Vol is the volume of liquid (L).

**Fig. 8:**
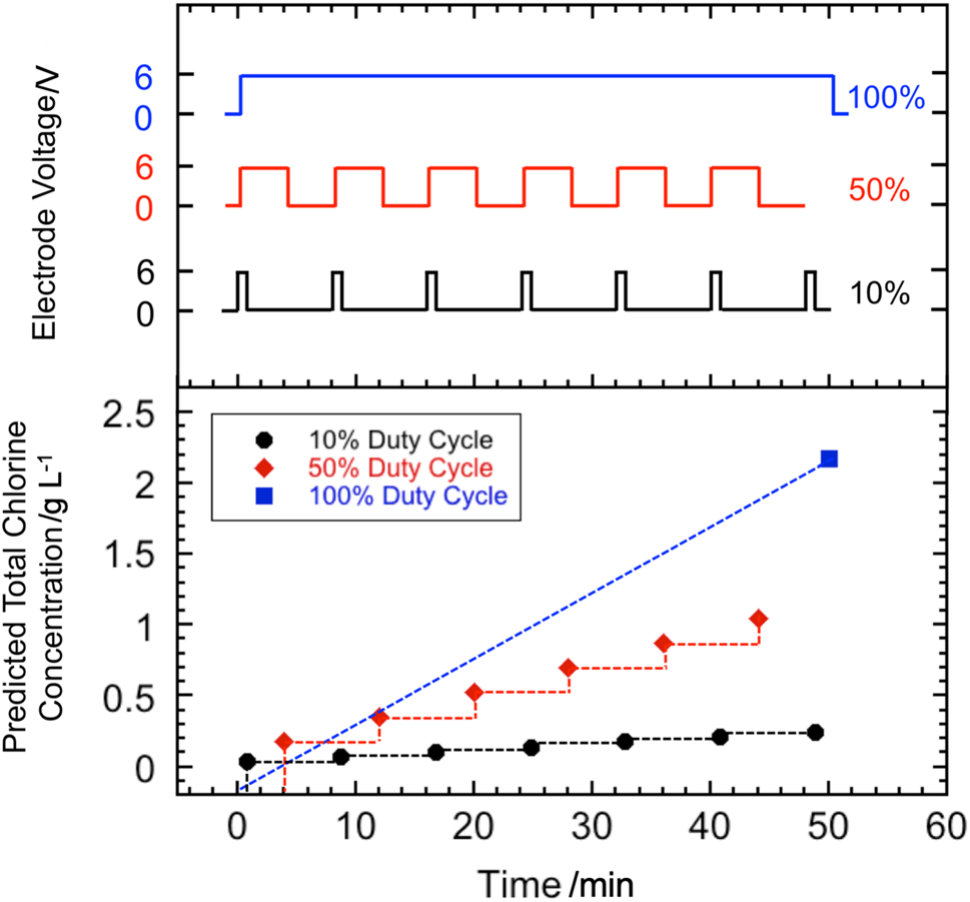
Theoretical chlorine concentration versus time for 10% duty cycle, 50% duty cycle, and continuous ON modes at 6 V

This formula assumes that all the applied charge is used for chlorine generation alone. From this, the theoretical value of chlorine concentration was calculated assuming that all of the applied charge was used for chlorine evolution. These were calculated for different duty cycles of the pulsed mode assuming the same total period of 8 min. Figure 8 shows this theoretical chlorine concentration versus time for 10% duty cycle, 50% duty cycle, and continuous ON modes at 6 V.

From Fig. 8, it is clear that the rate of generation of chlorine is proportional to the duty cycle. Thus, the chlorine generation rate was the highest for the continuous ON mode. The 50% duty cycle mode with a total ON time of 20 min would generate more chlorine than a 10% duty cycle mode with a total ON time of 10 min. However, we observed that disinfection was still achieved in the 10% duty cycle mode while less energy was consumed. Thus, the savings in disinfection energy is most likely attributed to the increased contact time with the *E. coli*.

### Prediction of disinfection energy for varying duty cycles

**Fig. 9:**
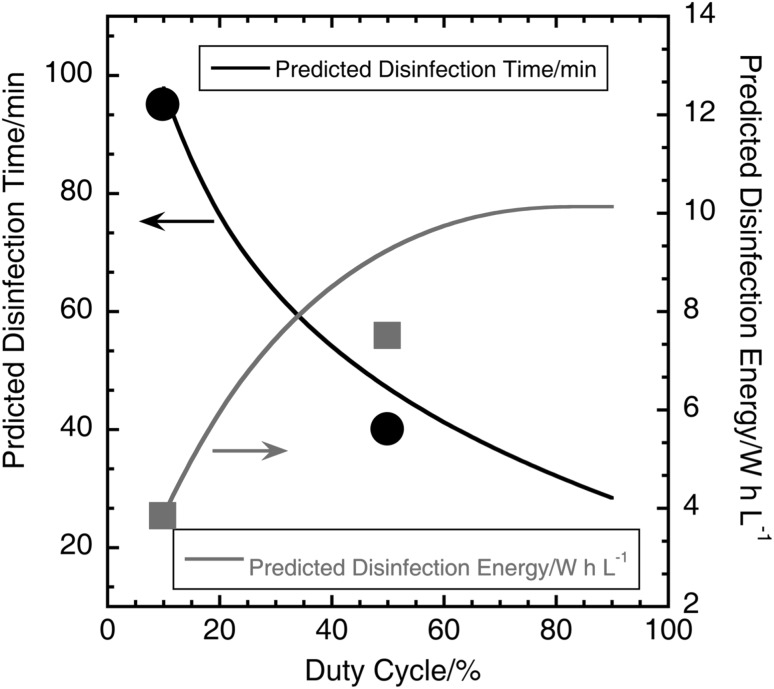
Plot of predicted disinfection time and energy versus duty cycle at 6 V. The blue circle and dot are the actual disinfection time and energy, respectively, for 10% duty cycle mode and the green circle and dot are the actual disinfection time and energy, respectively, for 50% duty cycle mode

From the calculations in the previous section and the experimental data, it was seen that the rate of generation of chlorine increased and the energy and total time required for disinfection decreased with increasing duty cycle. The disinfection time at 6 V for duty cycles varying from 10 to 90% was predicted from the continuous ON, 10% and 50% duty cycle experimental data by fitting a logarithmic function to the plot of disinfection time versus duty cycle. Disinfection energy was then calculated from the disinfection time, electrode voltage, and electrode current from Eq. 1. Figure 9 shows the plot of predicted disinfection time and energy versus duty cycles at 6 V. With increasing duty cycle, the time for disinfection decreases and disinfection energy increases, indicating a trade-off between disinfection energy and time as a function of duty cycle at 6 V. This trend is also expected for pulsed mode operation at other electrode voltages. The graph in Fig. 9 can be used to select an appropriate duty cycle based on application constraints, such as total volume of liquid to be processed per day and total energy available for disinfection.

## Conclusion

In conclusion, it was demonstrated that the energy required for electrochemical disinfection can be greatly reduced by pulsing the applied voltage. Pulsing allows for mixing of oxidants and improved contact time with the pathogens during the OFF time of the pulse and results in more energy-efficient disinfection compared to the continuous ON mode. This conclusion should also apply to co-generated oxidants such as hydroxyl radical and ozone. With a 10% duty cycle at 6 V, a 68% energy saving compared to the continuous ON mode at the same voltage. The energy saving is believed to be primarily due to improved contact time with the *E. coli* for a given concentration of chlorine. Further research is necessary to investigate the role of additional oxidants in the electrochemical disinfection of human liquid waste.
